# Case report: a precancerous lesion associated with HPV in the anal canal diagnosed by magnifying endoscopy with narrow-band imaging and resected by endoscopic submucosal dissection

**DOI:** 10.3389/fmed.2023.1103182

**Published:** 2023-04-26

**Authors:** Hengcun Li, Xiujing Sun, Ling Yang, Rui Xu, Peng Li

**Affiliations:** ^1^Department of Gastroenterology, Beijing Friendship Hospital, Capital Medical University, Beijing Key Laboratory for Precancerous Lesion of Digestive Disease, National Clinical Research Center for Digestive Disease, Beijing Digestive Disease Center, Beijing, China; ^2^Department of Gastroenterology, Beijing Fengtai Hospital, Beijing, China; ^3^Department of Pathology, Beijing Friendship Hospital, Capital Medical University, Beijing, China

**Keywords:** anal canal, HSIL, ME-NBI, ESD, case report

## Abstract

Although anal cancer remains rarely diagnosed in the world, its frequency is rising, especially in high-risk groups. The prognosis of advanced anal cancer is poor. However, there are still few reports on the endoscopic diagnosis and treatment of early anal cancer and its precancerous lesions. A 60-year-old woman was referred to our hospital for endoscopic treatment of a flat precancerous lesion in the anal canal, which was identified by narrow-band imaging (NBI) and confirmed by pathological examination in another hospital. The pathological results showed a high-grade squamous intraepithelial lesion (HSIL) in the biopsy specimen, and immunochemistry staining showed P16 positive, suggesting HPV infection. We performed pre-resection endoscopic examination for the patient. A lesion with a clear margin and tortuous dilated vessels was revealed under magnifying endoscopy with NBI (ME-NBI), which stayed unstained after iodine spraying. The lesion was successfully removed en bloc using ESD without complications, and the resected specimen was a low-grade squamous intraepithelial lesion (LSIL) with positive immunochemistry staining of P16. The patient underwent follow-up coloscopy a year after ESD, and the anal canal healed well with no suspicious lesions found. From this case, we can learn that ESD is safe and effective for curative resection of precancerous lesions of the anal canal.

## Introduction

Anal cancer principally comprises two morphologic variants: squamous cell carcinoma (SCC) and adenocarcinoma, with SCC comprising nearly 85% of all cases (1). In the general population, the age-standardized incidence rates are between 1 and 2 per 10,000 per year, with the incidence of SCC increasing by 1–3% per year in developed countries (2). The standard therapy is chemoradiation in Europe and the United States (3), with few cases diagnosed in an early stage. Although there are developments in the field of endoscopy, anal SCC and precancerous lesions are still not familiar to most gastroenterologists. There are a few risk factors of SCC of the anal canal, including human papillomavirus (HPV) infection and human immunodeficiency virus (HIV) infection (1). HPV infection is a precursor of cervical SCC and causes the progression from intraepithelial neoplasia to cancer (4). Evidence also showed the association between HPV infection and anal SCC, and a study showed that 88% of patients with anal cancer were diagnosed with HPV infection (5).

ME-NBI facilitates the clear visualization of superficial microvascular structure, which helps the identification of early cancer and precancerous lesions in the pharynx and esophagus (6, 7), as well as anal SCC and precancerous lesions (8). ESD is commonly used in the treatment of lesions in the digestive tract including the colorectum, but its application on anal SCC and precancerous lesions is rarely reported.

## Case presentation

**Figure 1:**
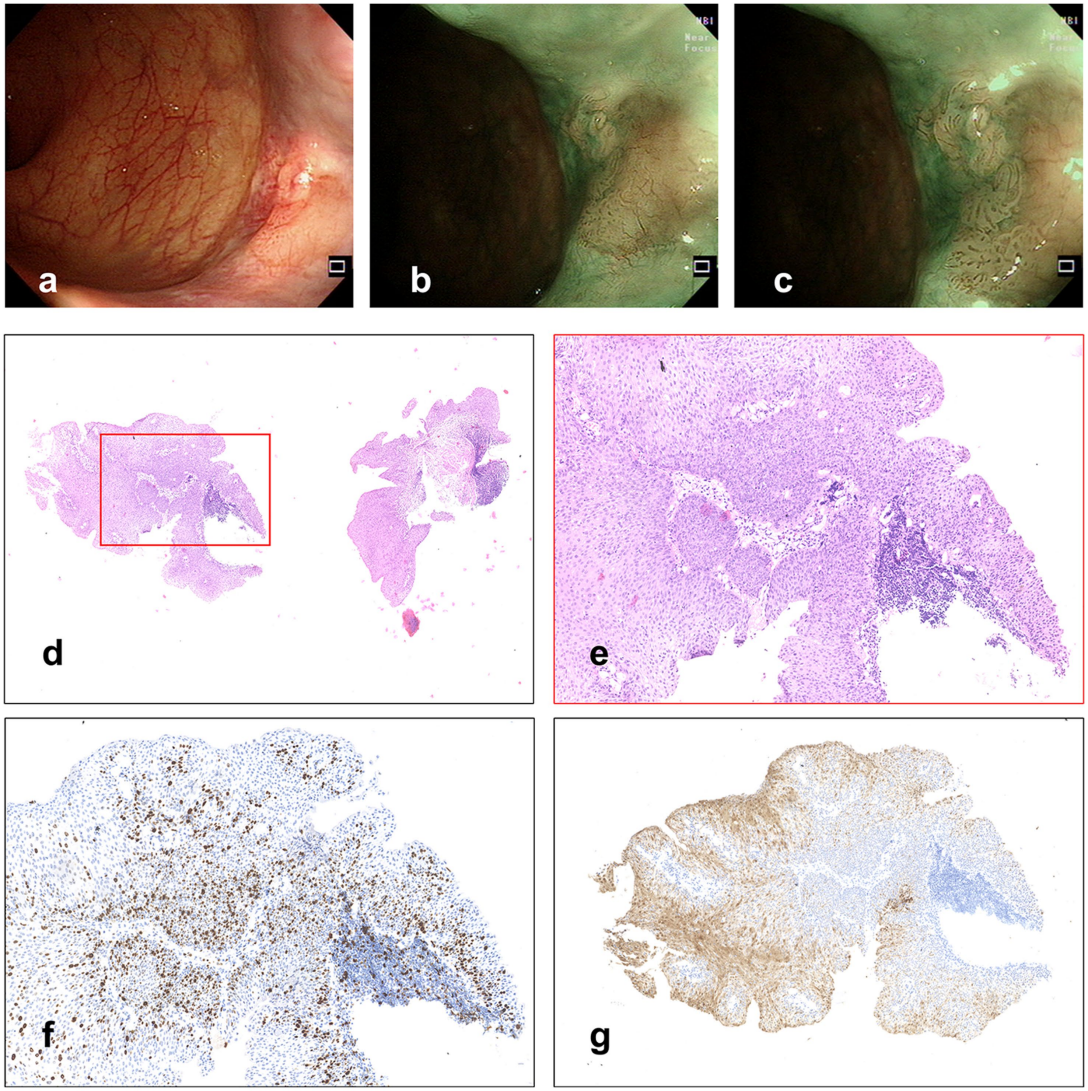
**(A)** A whitish superficial flat lesion with scattered reddish spots was discovered with WLI. **(B,C)** Lesion observed under NBI. **(D,E)** Hematoxylin and eosin staining of biopsy specimens, and **(F)** Immunochemistry stained by Ki-67, in which the Ki-67 positive proliferating cells are more than half of the epithelial layer. **(G)** Immunochemistry stained by P16.

A 60-year-old woman underwent colonoscopy because of changes in bowel habits in another hospital, with anal discomfort occasionally, without any other medical, family, or psychosocial history, and she claimed to be physically healthy before. When observing the anal canal using white light, a whitish superficial flat (0-IIb) lesion with scattered reddish spots was discovered (Figure 1A). In NBI without magnification, a 6 × 5 mm well-demarcated brownish lesion with dark brown dots was identified (Figures 1B,C). The biopsy was pathologically diagnosed to be HSIL (Figures 1D,E), with Ki-67 positive proliferating cells in more than half of the epithelial layer (Figure 1F) and P16 protein positive in immunochemistry (Figure 1G), which would have a good prognosis if successfully resected.

The patient was referred to our hospital for further examination and treatment. The temperature was 36.2°C, and other vital signs were normal on admission. The physical examination showed no significant abnormalities. The total cholesterol was 5.30 mmol/L (reference range 3.90–5.20 mmol/L), and other laboratory results were normal. The abdominal and pelvic CT scan (plain and enhanced scan) showed multiple cysts of the liver, with no suspicious malignant lesions found.

**Figure 2:**
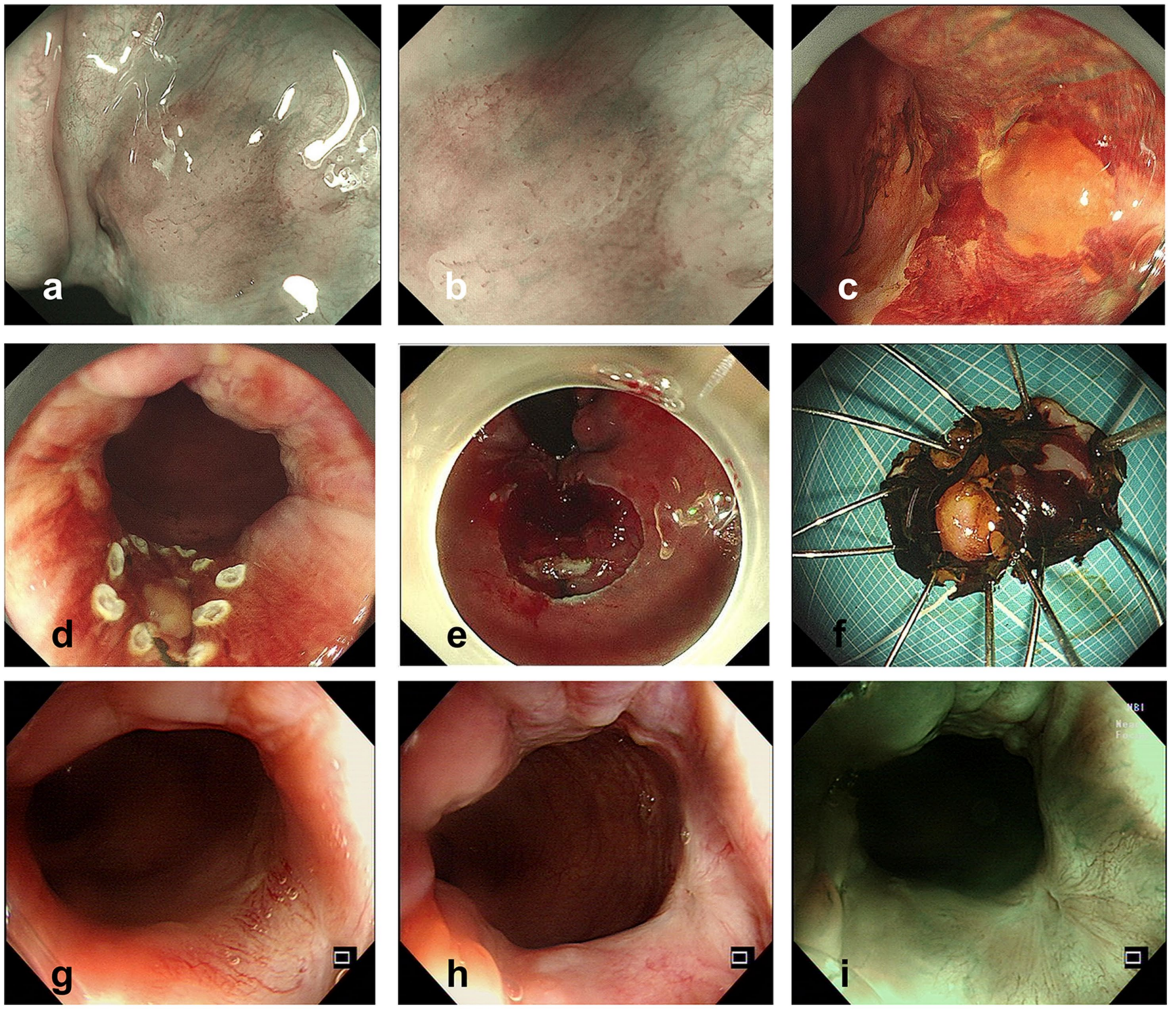
**(A,B)** Lesion observed with NBI **(A)** and ME-NBI **(B)** before ESD. **(C)** Lesion observed after iodine spraying. **(D,E)** Operation procedure of ESD. **(F)** Specimen resected by ESD (after iodine spraying). **(G–I)** Colonoscopy follow-up pictures 1 year after ESD.

**Figure 3:**
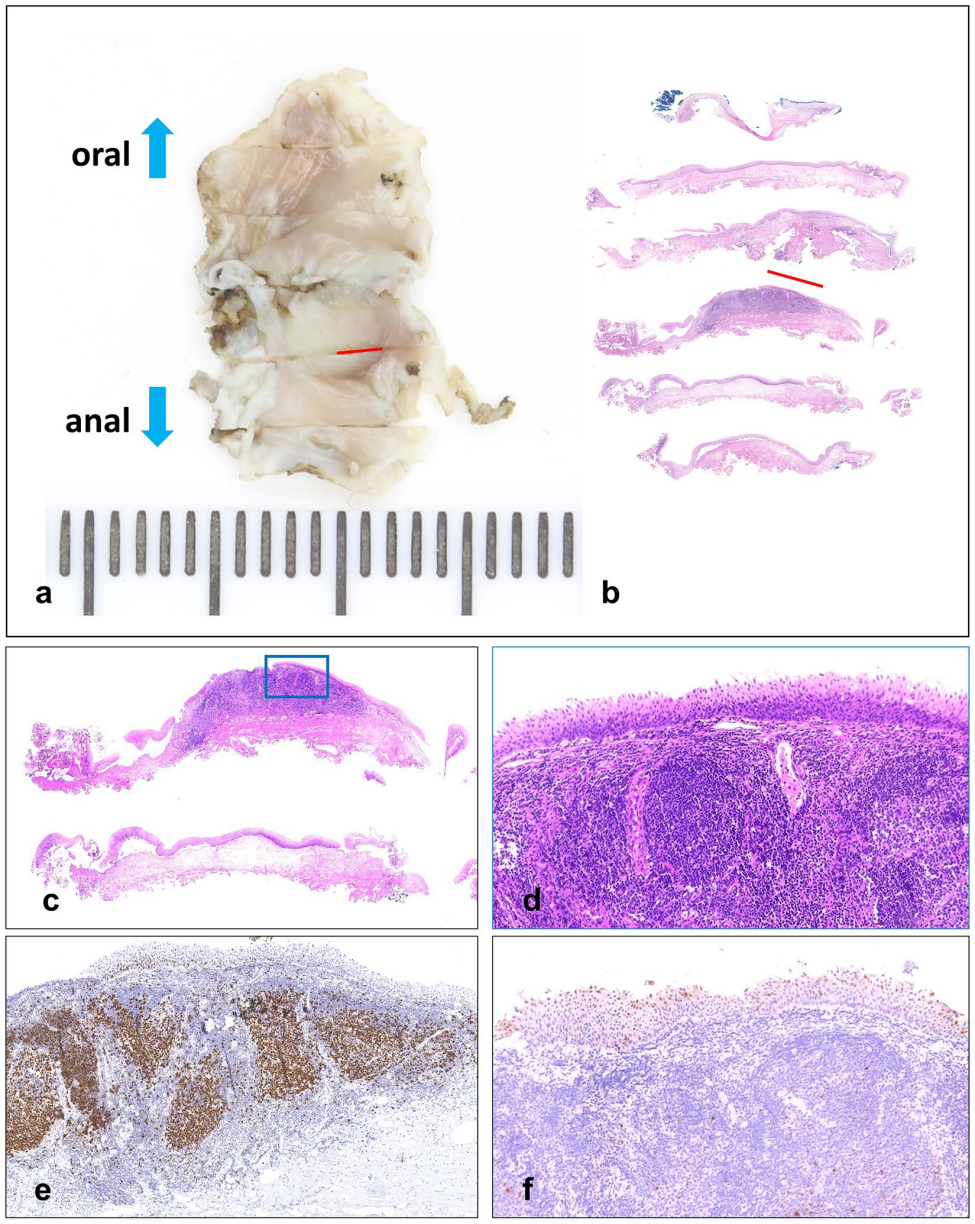
**(A,B)** Resected specimen (16 × 9 mm) sectioned at 2 mm intervals, and the red lines indicate LSIL areas. **(C,D)** Hematoxylin and eosin staining. **(E)** Immunochemistry stained by Ki-67, in which the Ki-67 positive proliferating cells are less than half of the epithelial layer. **(F)** Immunochemistry stained by P16.

The ME-NBI endoscopy was conducted, and we found a lesion with a clear margin and tortuous dilated vessels usually seen in early SCC of the esophagus (Figures 2A,B). After iodine spraying, the lesion stayed to be unstained, and the margin could be seen more clearly (Figure 2C). ESD was performed to resect the lesion en bloc (Figures 2D–F). The procedure was successful with no complication occurred. The resected specimen (16 × 9 mm) was sectioned at 2 mm intervals (Figure 3A), and the pathological examination revealed that the specimen resected was LSIL (Figures 3B–D) with Ki-67 positive proliferating cells less than half of the epithelial layer (Figure 3E) and P16 protein positive in immunochemistry (Figure 3F), with both vertical and horizontal margins free of tumor.

**Figure 4:**
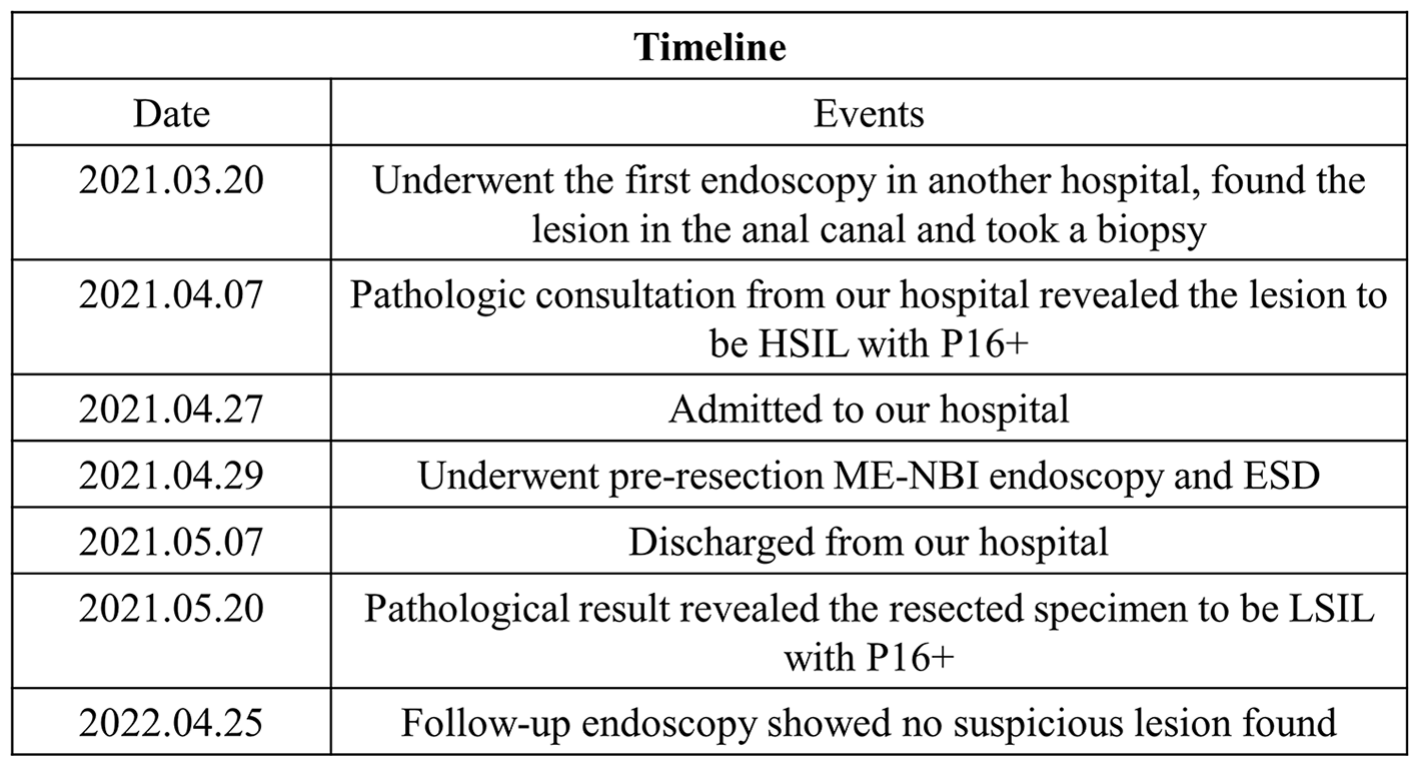
Timeline and corresponding events.

After 1 year of the endoscopic treatment, colonoscopy follow-up was performed, and no suspicious lesions were found (Figures 2G–I)， and the abdominal enhanced CT follow-up revealed no suspicious malignant lymph nodes or lesions. The timeline and corresponding events of the patient’s diagnosis and treatment process are displayed in Figure 4.

## Discussion

Anal cancer is an uncommon disease occurring in middle-aged adults, making up only 4% of cancers of the lower gastrointestinal tract (9). Anal cancers are rarely diagnosed at early stages, and the prognosis of advanced anal cancers is poor (10). SCC is the main pathological type of anal cancer, with well-recognized precursor lesions linked to HPV infection (11).

Although there are no guidelines for the screening of anal cancer, it is reasonable to screen high-risk populations with physical examination, anal cytology, and high-resolution anoscopy (HRA). With the increasing acceptance of endoscopy by the public, screening by colonoscopy should be noticed. There have been several case reports on the use of colonoscopy in the diagnosis and treatment of early cancer of the anal canal (12–15), but there is only one case report on the endoscopic diagnosis and treatment of anal HSIL (16), and it did not involve the association between anal canal HSIL and HPV infection. Histopathologic changes of lower anogenital squamous lesions are reported on the basis of a two-tiered system (The Lower Anogenital Squamous Terminology, LAST): a low-grade (LSIL) or high-grade (HSIL) squamous intraepithelial lesion (17) and the immunostaining of P16 is a biomarker for HPV-related precancerous lesions (11). The positive P16 immunostaining has been found to predict LSIL to HSIL progression and further to become SCC (17). Rates of progression of HSIL to anal SCC varied by population and risk factors, and estimates of 5-year anal SCC risk after HSIL diagnosis ranged from 3 to 32% (18). However, we should have a more comprehensive understanding of P16. P16 acts as a tumor suppressor protein (19), and research by Stefan Alexander Koerber et al. revealed that patients with HPV-DNA-positive and P16-positive tumors had significantly better overall survival after definitive chemoradiation, whereas p16 positive alone conferred no significant survival benefit (20). Therefore, more studies are needed to further explore the association between HPV infection and the occurrence, progression, and prognosis of anal canal cancer.

In the aspect of diagnosis, ME-NBI helps to identify suspicious lesions because the abnormal vasculature presenting dilatation, tortuosity, caliber change, and various shapes is similar to the intrapapillary capillary loop patterns of intraepithelial squamous cell carcinoma of the esophagus (21). In our case, a lesion with a clear margin and tortuous dilated vessels was identified under ME-NBI, and after iodine spraying, the lesion presented an unstained status, just like early cancers of the esophagus. There is currently no consensus on the management of anal HSIL, and it is reasonable to resect the lesion by ESD for it can achieve an en bloc resection with few complications, and several cases have been reported on the endoscopic treatment of anal early cancer or precancerous lesions, as mentioned above. Our procedure of ESD was successful, colonoscopy follow-up presented a good result. However, we should notice that the ESD procedure in the anal canal is more difficult because there are more vessels and nerves, which put forward higher technical requirements for the operator. In this case, the patient was under general anesthesia, and we treated the vessels with hemostatic forceps during operation carefully. After the resection procedure, we further treated the wound with argon plasma coagulation (APC) and then the hemostatic powder was sprayed to further prevent postoperative bleeding. In our opinion, when the patient feels pain, appropriate analgesic measures can be applied. The strength of the endoscopic treatment is minimally invasive, but the limitation is that preoperative evaluation is critical and may require surgical intervention for deeply invasive tumors.

The specimen resected by ESD was confirmed by pathological examination to be LSIL, which was not consistent with the result of the biopsy specimen. However, it was explainable because the lesion was less than 1 cm, and the biopsy might have resected the HSIL part of the lesion. Considering the HPV infection status, further treatment by ESD is necessary for the prevention of anal SCC.

## Conclusion

In conclusion, the precancerous lesions associated with HPV infection can progress to SCC, and the screening of early anal cancer and precancerous lesions is necessary. With the development and widespread of colonoscopy, a gastroenterologist should pay attention to the screening of anal lesions, especially with the help of ME-NBI. For early cancer and precancerous lesions with HPV infection in the anal canal, ESD is safe and effective for curative resection.

## Patient perspective

When asked about opinion on the endoscopic diagnostic and treatment process, the patient commented that endoscopic treatment is less traumatic and has a quick recovery, with endoscopy follow-up showing a complete cure, which was very friendly and efficient for patients. We agreed that the minimally invasive and effective operation and regular follow-up colonoscopy can bring more benefits to patients.

## Data availability statement

The original contributions presented in the study are included in the article/Supplementary material, further inquiries can be directed to the corresponding author.

## Ethics statement

The studies involving human participants were reviewed and approved by The Ethics Committee of Beijing Friendship Hospital, Capital Medical University. The patients/participants provided their written informed consent to participate in this study. Written informed consent was obtained from the individual(s) for the publication of any potentially identifiable images or data included in this article.

## Author contributions

HL wrote the manuscript. PL and XS designed the study and supervised the manuscript. HL, LY, and RX collected the clinical data. PL, XS, and HL participated in the diagnosis and treatment of the patient. All authors contributed to the article and approved the submitted version.

## Funding

This study was supported by the National Natural Science Foundation of China (82070575) and Capital’s Funds for Health Improvement and Research (2020–2-2026).

## Conflict of interest

The authors declare that the research was conducted in the absence of any commercial or financial relationships that could be construed as a potential conflict of interest.

## Publisher’s note

All claims expressed in this article are solely those of the authors and do not necessarily represent those of their affiliated organizations, or those of the publisher, the editors and the reviewers. Any product that may be evaluated in this article, or claim that may be made by its manufacturer, is not guaranteed or endorsed by the publisher.

## References

[1] Nelson VM Benson AB 3rd. Epidemiology of Anal Canal cancer. Surg Oncol Clin N Am. (2017) 26:9–15. doi: 10.1016/j.soc.2016.07.001 27889039

[2] Grulich AE Poynten IM Machalek DA Jin F Templeton DJ Hillman RJ. The epidemiology of anal cancer. Sex Health. (2012) 9:504–8. doi: 10.1071/SH12070 22958581

[3] Joseph DA Miller JW Wu X Chen VW Morris CR Goodman MT. Understanding the burden of human papillomavirus-associated anal cancers in the US. Cancer. (2008) 113:2892–900. doi: 10.1002/cncr.23744, PMID: 18980293 PMC2729501

[4] Fenger C. Anal neoplasia and its precursors: facts and controversies. Semin Diagn Pathol. (1991) 8:190–201. PMID: 1656504

[5] Frisch M Glimelius B van den Brule AJ Wohlfahrt J Meijer CJ Walboomers JM. Sexually transmitted infection as a cause of anal cancer. N Engl J Med. (1997) 337:1350–8. doi: 10.1056/NEJM199711063371904 9358129

[6] Nakanishi H Doyama H Takemura K Yoshida N Tsuji K Takeda Y. Detection of pharyngeal cancer in the overall population undergoing upper GI endoscopy by using narrow-band imaging: a single-center experience, 2009-2012. Gastrointest Endosc. (2014) 79:558–64. doi: 10.1016/j.gie.2013.09.023 24246793

[7] Muto M Minashi K Yano T Saito Y Oda I Nonaka S. Early detection of superficial squamous cell carcinoma in the head and neck region and esophagus by narrow band imaging: a multicenter randomized controlled trial. J Clin Oncol. (2010) 28:1566–72. doi: 10.1200/JCO.2009.25.4680, PMID: 20177025 PMC2849774

[8] Morisaki T Isomoto H Akazawa Y Yamaguchi N Ohnita K Takeshima F. Beneficial use of magnifying endoscopy with narrow-band imaging for diagnosing a patient with squamous cell carcinoma of the anal canal. Dig Endosc. (2012) 24:42–5. doi: 10.1111/j.1443-1661.2011.01153.x, PMID: 22211411

[9] Clark MA Hartley A Geh JI. Cancer of the anal canal. Lancet Oncol. (2004) 5:149–57. doi: 10.1016/S1470-2045(04)01410-X 15003197

[10] Gerard JP Chapet O Samiei F Morignat E Isaac S Paulin C. Management of inguinal lymph node metastases in patients with carcinoma of the anal canal: experience in a series of 270 patients treated in Lyon and review of the literature. Cancer. (2001) 92:77–84. doi: 10.1002/1097-0142(20010701)92:1<77::AID-CNCR1294>3.0.CO;2-P, PMID: 11443612

[11] Fléjou JF. An update on anal neoplasia. Histopathology. (2015) 66:147–60. doi: 10.1111/his.12574 25283345

[12] Horimatsu T Miyamoto S Ezoe Y Muto M Yoshizawa A Sakai Y. Education and gastrointestinal imaging: case of early-stage squamous cell carcinoma of the anal canal diagnosed using narrow-band imaging system with magnification. J Gastroenterol Hepatol. (2012) 27:1406. doi: 10.1111/j.1440-1746.2012.07182.x, PMID: 22823917

[13] Tsuji S Doyama H Yamada S Tominaga K Ota R Yoshikawa A. Endoscopic submucosal dissection of a squamous cell carcinoma in situ in the anal canal diagnosed by magnifying endoscopy with narrow-band imaging. Clin J Gastroenterol. (2014) 7:233–7. doi: 10.1007/s12328-014-0481-7, PMID: 26183742

[14] Ito T Morita S Shimeno N Uehara K Imai Y Inokuma T. The prospect of endoscopic submucosal dissection for early anal canal squamous cell carcinoma. Clin J Gastroenterol. (2016) 9:384–8. doi: 10.1007/s12328-016-0690-3, PMID: 27738909

[15] Chou YP Saito Y Matsuda T Nakajima T Mashimo Y Moriya Y. Novel diagnostic methods for early-stage squamous cell carcinoma of the anal canal successfully resected by endoscopic submucosal dissection. Endoscopy. (2009) 41:E283–5. doi: 10.1055/s-0029-1214942, PMID: 19866431

[16] Kasuga K Saito Y Wu SYS Takamaru H Sakamoto T Sekine S. Impact of endoscopic submucosal dissection of an anal squamous intraepithelial lesion with indistinct border. Endoscopy. (2020) 52:E75–7. doi: 10.1055/a-0977-2446, PMID: 31529437

[17] Bull-Henry K Morris B Buchwald UK. The importance of anal cancer screening and high-resolution anoscopy to gastroenterology practice. Curr Opin Gastroenterol. (2020) 36:393–401. doi: 10.1097/MOG.0000000000000661, PMID: 32701604

[18] Faber MT Frederiksen K Palefsky JM Kjaer SK. Risk of anal cancer following benign anal disease and anal cancer precursor lesions: a Danish Nationwide cohort study. Cancer Epidemiol Biomark Prev. (2020) 29:185–92. doi: 10.1158/1055-9965.EPI-19-0601, PMID: 31597665

[19] Weinberger PM Yu Z Haffty BG Kowalski D Harigopal M Brandsma J. Molecular classification identifies a subset of human papillomavirus--associated oropharyngeal cancers with favorable prognosis. J Clin Oncol. (2006) 24:736–47. doi: 10.1200/JCO.2004.00.3335, PMID: 16401683

[20] Koerber SA Schoneweg C Slynko A Krug D Haefner MF Herfarth K. Influence of human papillomavirus and p16(INK4a) on treatment outcome of patients with anal cancer. Radiother Oncol. (2014) 113:331–6. doi: 10.1016/j.radonc.2014.11.013, PMID: 25465729

[21] Yoshida T Inoue H Usui S Satodate H Fukami N Kudo SE. Narrow-band imaging system with magnifying endoscopy for superficial esophageal lesions. Gastrointest Endosc. (2004) 59:288–95. doi: 10.1016/S0016-5107(03)02532-X, PMID: 14745410

